# Effects of Aspirin on Kidney Biopsy Bleeding Complications: A Systematic Review and Meta-Analysis (PROSPERO 2021 CRD42021261005)

**DOI:** 10.34067/KID.0000000000000091

**Published:** 2023-03-23

**Authors:** Miguel Relvas, Joana Gonçalves, Inês Castro, Hugo Diniz, Luís Mendonça, Luís Coentrão

**Affiliations:** 1 Nephrology Department, Centro Hospitalar Universitário de São João, Porto, Portugal; 2 Department of Medicine, Faculty of Medicine, University of Porto, Porto, Portugal; 3 Department of Surgery and Physiology, UnIC@RISE, Faculty of Medicine of the University of Porto, Porto, Portugal; 4 Nephrology & Infectious Diseases R&D, i3S—Institute for Research & Innovation in Health, Porto, Portugal

**Keywords:** Clinical Nephrology, aspirin, bleeding, image-guided biopsy, kidney, meta-analysis, percutaneous biopsy

## Abstract

Postprocedural bleeding is the main complication of percutaneous kidney biopsy (PKB). Therefore, aspirin is routinely withheld in patients undergoing PKB to reduce the bleeding risk. The authors aimed to examine the association between aspirin use and bleeding during PKB. This systematic review and meta-analysis was performed according to the Preferred Reporting Items for Systematic Reviews and Meta-Analyses guidelines. The article search was performed on MEDLINE and Scopus using queries specific to each database. Article inclusion was limited to primary studies. The meta-analysis compared the risk of major bleeding events between the aspirin-exposed versus nonexposed group. Pooled effect estimate was examined using random effects presented as odds ratio with 95% confidence intervals. Heterogeneity was assessed through Cochrane I^2^ test statistics. Sensitivity and subgroup analyses were also performed according to kidney type. Ten studies were included in the review and four studies were included in the meta-analysis, reviewing a total of 34,067 PKBs. Definitions for significant aspirin exposure were inconsistent between studies, limiting comparisons. Studies with broader definitions for aspirin exposure mostly showed no correlation between aspirin use and postbiopsy bleeding. Studies with strict definitions for aspirin exposure found an increased risk of hemorrhagic events in the aspirin-exposed group. No significant differences were found between the aspirin-exposed and comparison groups regarding major bleeding events (odds ratio 1.72; 95% confidence interval 0.50 to 5.89, I^2^=84%). High-quality evidence on the effect of aspirin on the bleeding risk is limited. Our meta-analysis did not show a significantly increased risk of major bleeding complications in aspirin-exposed patients. Further studies are needed to define a more comprehensive approach for clinical practice.

## Introduction

Before *Iversen* and *Brun*'s initial description of kidney biopsy in the 1950s,^1^ clinical and analytical findings were the basis for diagnosing kidney diseases.^2^ Currently, kidney biopsies are performed in both native and graft kidneys, playing a crucial role in diagnosing many primary and secondary renal conditions.^3,4^

Image guidance and automated biopsy devices have improved the diagnostic yield of kidney biopsies while simultaneously minimizing associated complications.^1,3–6^ Despite advancements in the technique and equipment, major hemorrhagic complications are still responsible for lengthier hospital stays and increased treatment costs,^6,7^ occurring in 0.24%–6.6% of all patients subjected to kidney biopsies.^2,4,6–23^

Various factors have been associated with increased biopsy complication rates, including unmodifiable factors, such as age and sex, comorbidities (high serum creatinine levels, obesity, anemia, systemic autoimmune diseases, amyloidosis), and procedure-associated aspects.^2,5–7,10,23–25^ Most centers have similar safety requirements, with universal consensus regarding the need for adequate blood pressure control and correction of any preexisting coagulopathy.^26,27^ Anticoagulants and antiplatelet drugs constitute other potential risk factors of hemorrhagic complications. Strategies regarding their preprocedural cessation are highly heterogeneous.

Aspirin has historically been considered a contraindication for percutaneous kidney biopsy (PKB). As one of the most widely prescribed antiaggregants worldwide,^28–30^ its use is a common hurdle in patients proposed for PKB and determining whether and when to stop this drug before a biopsy assumes extreme relevance. Classical recommendations for a 7 to 10-day washout period were based on the premise that aspirin permanently inactivates cyclooxygenase, a key platelet enzyme for the synthesis of thromboxane.^31^ A daily dose of 30 mg aspirin is sufficient to completely suppress thromboxane production within 1 week.^32^ This can only be reversed by the generation of new platelets that have a mean life span of approximately 10 days.^33^

Various entities have already debated this issue; however, a standardized and validated approach is still unavailable. The Society of Interventional Radiology recommends halting aspirin use at least 5 days before the biopsy.^27^ The Canadian Cardiovascular Society recommends ceasing aspirin 7–10 days before the intervention.^34^ The American College of Chest Physicians also suggests aspirin cessation 7–10 days before the intervention in low cardiovascular risk patients, but recommends maintaining aspirin use in moderate-to-high cardiovascular risk patients.^35^ The Kidney Health Australia Caring for Australasians with Renal Impairment guidelines support aspirin use in high cardiovascular risk patients, but propose a late withdrawal strategy (3–7 days before the biopsy) in low-risk patients.^36^

This article aims to review original articles regarding the influence of aspirin on the bleeding risk of patients subjected to PKB to help establish a standardized preprocedural strategy for its cessation.

## Methods

This systematic review and meta-analysis was registered on PROSPERO (CRD42021261005) and is reported according to the Preferred Reporting Items for Systematic Reviews and Meta-Analyses guidelines.

### Study Inclusion Criteria

#### Types of Studies and Study Participants

Randomized controlled trials and observational studies were considered eligible if they reported bleeding complications after kidney biopsy in patients under aspirin exposure. Case reports, narrative or systematic reviews, and meta-analyses were excluded. Studies dealing exclusively with dual antiplatelet therapy were excluded. All study participants were eligible, regardless of age, sex, underlying comorbidities, the presence of native or graft kidneys, or indications for kidney biopsy.

#### Types of Interventions

Studies were considered eligible for selection if kidney biopsies were performed percutaneously under image guidance: ultrasound (US) or computed tomography. Studies that included kidney biopsies performed using transjugular or other nonpercutaneous approaches were excluded.

#### Types of Outcome Measures

Bleeding complications comprised the primary outcomes. Major bleeding complications were defined as those resulting in permanent adverse sequelae or death or those requiring interventional therapy—medical (including blood transfusions), radiological, or surgical (such as embolization or coiling). Minor complications were defined as hematuria or hematomas requiring no active intervention or prolonged hospitalization and other nonsevere complications.

#### Data Source

**Figure 1:**
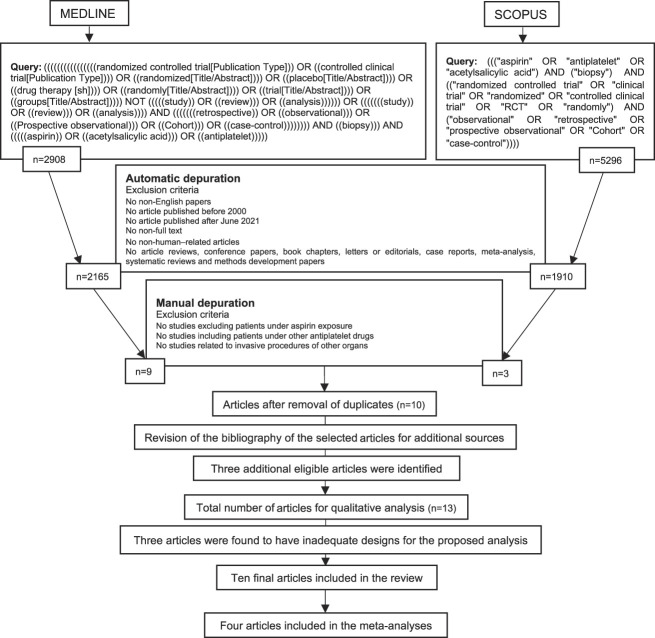
Breakdown of obtained articles from the initial search included in the review, including queries used for each database.

Two different electronic databases were used, MEDLINE and Scopus. The following medical subject heading terms were used to identify potential articles: ”aspirin,” “acetylsalicylic acid,” and “antiplatelet” (Figure 1). Each medical subject heading term was then combined with “biopsy.” The authors agreed on a set of inclusion and exclusion criteria for automatic depuration. The search criteria were coded into queries specific to each database. The article search was conducted between July and August 2021. All studies published until the final article search were considered for inclusion.

### Study Selection

Two investigators (JG and MR) independently evaluated the articles to determine their suitability for inclusion in this review. Eligible articles had their bibliographies examined to identify other relevant studies for this systematic review. Disagreements between the two reviewers were resolved by a third reviewer (LC).

### Data Collection Process and Data Items

The following information—study type, type of kidney (native versus graft), number of kidney biopsies, type of image guidance, number of kidney biopsies performed in patients using aspirin, last known intake and dosage of the drug, clinical outcome, odds ratio (OR), and major limitations—was extracted from each selected article (when applicable) and condensed in Table 1. One author (JG) extracted the data, and the other author (MR) checked the information retrieved. Divergences over the interpretation of data were resolved by team discussion.

**Table 1:** Study Characteristics.

Author (yr)	Study Type	Type of Kidney	No of Kidney Biopsies	Image Guiding Technique	No of Kidney Biopsies Performed in Patients Using Aspirin	Aspirin Cessation Date	Aspirin Dose	Clinical Outcome	OR	Major Limitations
Morgan *et al.*, 2015^11^	Observational retrospective cohort	Graft	235^a^	US	59	NR	NR	No statistically significant association was found between aspirin use and the occurrence of bleeding complications.	OR 0.65	Retrospective design. Analysis of only a subgroup of patients for specific characteristics. No data regarding aspirin cessation date and doses used.
Baffour *et al.*, 2017^13^	Observational retrospective cohort	Graft	6700	US	1994	8–10 d: 664 biopsies 4–7 d: 855 biopsies 0–3 d: 475 biopsies	81 or 325 mg	325-mg dosage within 3 d of a biopsy was associated with an increased risk of bleeding. Other dosages and time interval exposure were not associated with an increase in the bleeding risk.	8–10 d: OR 0.405 4–7 d: OR 0.518 0–3 d: OR 2.52	Retrospective design. Arbitrary stratification of patient aspirin exposure into four groups may have confounded results. Possible higher level interaction effects between variables because of a low number of complications that limited the models used.
Kuiper *et al.*, 2017^18^	Observational prospective cohort	Graft	154	US	26	5 d: 25 biopsies Within 5 d: 1 biopsy	NR	Higher risk of a bleeding complication in patients who reported the use of aspirin.	OR 3.19	Only one cessation interval was evaluated. The patient who breached the aspirin cessation protocol was included in the same group for risk analysis. Small sample size.
Schwarz *et al.*, 2005^20^	Observational prospective cohort	Graft	1670	US	234	Sustained use	100 mg	Significant higher risk of gross hematuria. Nonsignificant increased risk for perirenal hematomas.	NR	No data regarding aspirin cessation dates for high-dose aspirin. Analysis for bleeding risk in aspirin-exposed patients was limited to those with sustained use at the time of the biopsy.
Mackinnon *et al.*, 2008^15^	Observational retrospective cohort	Native	1120	US	122	5 d: 54 biopsies Sustained use: 68 biopsies	NR	Higher risk of hemoglobin drop ≥1.0 g/dl (minor complication) in patients with concurrent antiplatelet therapy. No association was found with major complications.	NR	Retrospective design. Patients with exposures to different antiplatelet agents were pooled in the same group with no subanalysis regarding the bleeding risk associated with each specific drug.
Monahan *et al.*, 2019^16^	Observational retrospective cohort	Native	2204	US or CT	681	Within 10 d	81 or 325 mg	No significant associations between the variable and the outcome for any dosage.	325 mg: OR 0.778 Any dose: OR 0.612	Retrospective design. Exclusion of potentially clinically important complications below CTCAE 3. Low number of complications limited the models used. Potential confounding by periprocedural hypertension (not included in the regression model).
Lees *et al.*, 2017^17^	Observational retrospective cohort	Native	2563	US	327	Sustained use	75 mg	No increase in the risk of major bleeding nor the risk of hemoglobin drop >2 g/L or other morbidities in patients with concurrent aspirin use.	OR 0.6	Retrospective design. Small absolute number of complications. Underpowered, prospective confirmation would be required.
Atwell *et al.*, 2010^14^	Observational retrospective cohort	Native and graft	5832	US or CT	1270	Within 10 d	81 or 325 mg	No association was found between antiplatelet exposure and the risk of bleeding.	NR	Retrospective design. Exclusion of potentially clinically important complications below CTCAE 3. Use of a general database maintained over several years, with imprecise data regarding complications. Only one interval of aspirin exposure was evaluated.
Potretzke *et al.*, 2019^19^	Observational retrospective cohort	Native and graft	11,350	US or CT	NR	8–10 d or 4–7 d or 1–3 d or the day of the procedure	81 or 325 mg	Any aspirin usage within 3 d of the biopsy was associated with an increase in the risk of bleeding, particularly with a dosage of 325 mg. The association between taking aspirin on the day of the biopsy and the risk of bleeding complications was even more significant. Other time intervals for aspirin exposure were not associated with a higher risk of bleeding.	Renal biopsy (all) Any dose, day of biopsy: OR 12.4 325 mg, within 3 d: OR 9.57 81 mg, within 3 d: OR 2.1 Any dose, within 3 d: 3.66 Native kidney biopsy Any dose, day of biopsy: OR 5.75 325 mg, within 3 d: OR 5.57 Any dose, within 3 d: 5.51	Retrospective design. Potential confounders, such as underlying kidney or hepatic disease or use of other antithrombotic agents beyond clopidogrel and warfarin, were not considered. Aspirin usage data were dependent on patient self-reporting, which carries a risk of inaccuracy. Multiple biopsy operators with inherent variation in procedural techniques.
Bonani *et al.*, 2021^21^	Observational retrospective cohort	Native and graft	2239	US	37	Sustained use	NR	The risk to experience a major hemorrhage was higher in patients under aspirin.	OR 6.39	Retrospective design. Incomplete dataset. Patients under aspirin who stopped therapy a week before the biopsy were included in the same subgroup as those with no aspirin exposure.

NR, not reported; OR, odds ratio; US, ultrasound; CT, computed tomography; CTCAE, Common Terminology Criteria for Adverse Events.

a Although a total of 2514 biopsies were considered in this study, only 235 biopsies were included in the subgroup analysis that allowed the authors to study the relation between aspirin exposure and the development of bleeding complications.

### Assessment of the Risk of Bias in the Included Studies

The risk of bias was independently evaluated by two reviewers (JG, MR) using the Risk Of Bias In Non-randomized Studies of Interventions tool. Seven different bias domains were evaluated independently for each selected article—confounding bias, selection bias, classification bias, performance bias, missing data, measurement bias, and reporting bias. Disagreements were resolved by a third reviewer (LC) or consensus-based discussion.

### Data Analysis

We conducted a meta-analysis to quantitatively assess the risk of major bleeding complications between aspirin-exposed versus nonexposed groups. In the exposed group, we included PKB performed in patients with aspirin exposure within 3 days of biopsy. The nonexposed group included biopsies performed in patients with no aspirin exposure at least 7 days before this procedure. Meta-analyses were performed using Review Manager version 5.3 (Cochrane Collaboration). Dichotomous outcome results were expressed using OR with a 95% confidence interval (CI). A *P*-value of <0.05 was considered significant. Data were pooled using a random-effects model (Mantel-Haenszel method) to incorporate both within-study and between-study variances. Statistical heterogeneity among studies was assessed using I^2^ tests with substantial heterogeneity defined as I^2^ >50%.^37^ Sensitivity analyses were performed to explore potential sources of heterogeneity, including removal of studies with a non–low risk of bias for each domain assessed and the consecutive removal of each individual study. We also conducted a subgroup analysis according to kidney type (native or graft). Publication bias was assessed through visual inspection of the funnel plot.

## Results

### Study Selection

Figure 1 presents the study selection flow of this systematic review. In total, 8204 articles were identified through electronic searches in the two selected databases. Automatic depuration eliminated 4129 articles. Of the remaining 4075 articles, 4064 were found ineligible for inclusion after having their titles and abstracts screened. Two of the remaining articles were duplicates. After scanning the remaining ten articles' bibliographic references, three additional studies were found to fall in the scope of the review, meeting the requisited criteria. The methodology of each article was then assessed to determine suitability for our analysis. Three articles were found to have inadequate designs for the proposed analysis (Reschen *et al.*,^38^ Ori *et al.*^39^ and Trajceska *et al.*^12^). The remaining ten^11,13–21^ articles were included in our analysis. Four^11,13,17,21^ of the ten studies selected were also included in quantitative synthesis.

### Study Characteristics

Details of the included trials, such as study design, type of kidney biopsied (native versus graft), total number of biopsies included, number of biopsies performed under aspirin exposure, image technique used, criteria for aspirin exposure, aspirin doses used, and clinical outcomes related to bleeding complications, are provided in Table 1.

This systematic review included studies conducted between 2002 and 2019, with an aggregate 34,067 PKBs. Sample sizes ranged from 154 to 11,350 PKBs. Four studies related exclusively to kidney graft biopsies,^11,13,18,20^ three only included native kidney biopsies,^15–17^ and three others discussed complications in both native kidneys and grafts.^14,19,21^ All biopsies were performed under image guidance—in all but three studies (which also included computed tomography–guided biopsies),^14,16,19^ US was the only imaging modality used. All studies were observational cohort studies, and only two were prospective in nature.^18,20^

The definition used for aspirin exposure was disparate. While some studies limited valid aspirin exposure to a confirmed dose the same day as the kidney biopsy, others had broader criteria that included patients with any confirmed dose within 10 days of the intervention.^14,16^ In other studies, different time intervals were considered,^13,18,19^ whereas some did not objectively define the date of the last confirmed dose.^11^ The definition of bleeding complications was more consistent among the selected studies.

### Risk of Bias within and across Studies

**Figure 2:**
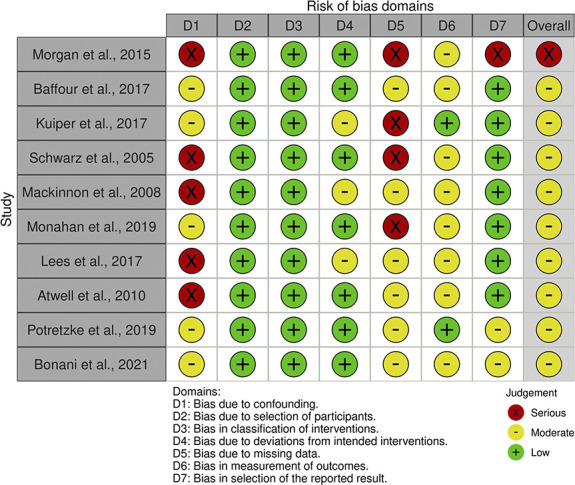
Risk of bias summary (Risk Of Bias In Nonrandomized Studies of Interventions tool).

**Figure 3:**
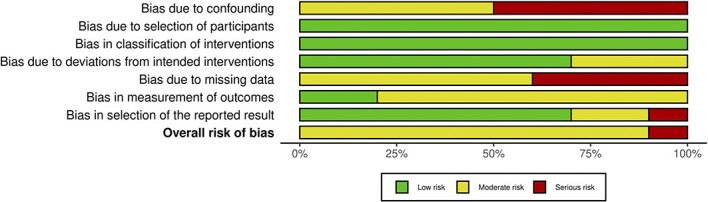
Risk of bias graph (Risk Of Bias In Nonrandomized Studies of Interventions tool).

The authors used the ROBINS-I tool to estimate the risk of bias of each included article across seven domains. The risk of bias assessments is presented in Figures 2 and 3. Most of the studies included in this review were not specifically designed to evaluate the correlation between aspirin use and bleeding complications. As such, in most of the included articles, solid data regarding the time frame of aspirin withdrawal is scarce and appropriate analysis for confounding factors is absent. Thus, all articles were found to be at moderate-to-serious risk of overall bias.

### Synthesis of Results

#### Kidney Graft Biopsies

A total of 11,038 biopsies were assessed in the articles related to kidney grafts, including elective (or surveillance) and indication biopsies.

Morgan *et al.*^11^ analyzed 2514 graft biopsies. A major complication of a graft biopsy was defined as the need for additional hospitalization time and/or an active intervention, including hospital admission, transfusion of blood products, interventional radiology vascular procedure, surgical exploration, transplant removal, and/or death. Minor complications were not evaluated in this study. Major complications occurred in 47 patients (1.9%). Although patients were advised to stop both anticoagulants and antiplatelet agents 7 days before the biopsy, that was not always feasible (particularly in the case of indication biopsies). To study the relationship between aspirin and the risk of biopsy complications, the authors selected all patients with a biopsy complication and a subset of patients without any complication using a 1:4 ratio, forming a subgroup of 235 individuals (47 patients with major complications and 188 patients with no complications). Nine patients met the article's aspirin exposure criteria in the complication-afflicted fraction (19.15%) while 50 patients had relevant aspirin intake in the uncomplicated fraction (26.60%). The percentages stated in the original article (15% versus 85% aspirin exposure in the complicated and uncomplicated subgroups respectively) were erroneously calculated using the total number of patients in the aspirin-exposed subgroup as the whole, meaning the percentages solely represent the 1:4 ratio selected for the construction of the control sample. Univariate analysis suggested aspirin use was not significantly associated with biopsy complications (including hemorrhagic phenomena) (see Table 1). In addition, the authors compared the risk of complications between indication biopsies (n=1599) and protocol biopsies (n=915). The first were associated with a significantly higher complication rate than the latter (2.7% versus 0.33%, *P*-value<0.001).

Baffour *et al.*^13^ analyzed the association between aspirin use, its dosage (81 or 325 mg), and the development of hemorrhagic complications in 6700 kidney graft biopsies. Most of the biopsies were performed in patients without aspirin exposure or with a last registered dose more than 10 days earlier (n=4706). Six hundred sixty-four biopsies were performed within 8 to 10 days of the last dose; 855 others within 4–7 days; and on 475 occasions, the patients had taken aspirin within 3 days of the procedure (n=475). The authors analyzed two outcomes: general and major bleeding complications, with events graded on the basis of the SIR Standards of Practice complication classification system (minor complications: A—requiring no therapy or consequence; B—requiring nominal therapy with no consequence; major complications: C—requiring therapy or minor hospitalization; D—requiring major therapy, unplanned increase in level of care, or prolonged hospitalization; E—resulting in permanent adverse sequelae; F—resulting in death). For both outcomes, adjusting for multiple testing, only a dosage of 325 mg within 3 days showed a significant association (OR=3.87, *P*-value=0.032 for any bleeding complication; OR=6.30, *P*-value=0.024 for major bleeding complication). However, aspirin in any dosage and up to 7 days before the biopsy showed a clear trend toward more major complications (*e.g.*, OR of 4.39, *P*=0.010 for aspirin in any dosage within 7 days of the biopsy). The authors also compared the risk of general and major bleeding complications between protocol biopsies (80.3%) and indication biopsies (19.7%). For both events, indication biopsies presented a statistically significantly higher risk (OR=2.27, *P*-value=0.012 and OR=6.84, *P*-value<0.001, respectively).

The prospective study by Kuiper *et al.*^18^ evaluated 154 transplanted patients who underwent graft biopsy. The authors aimed to determine whether bleeding tests could predict bleeding after kidney biopsy. Bleeding outcomes included hemoglobin drops of 1 mmol/L or more, positive US findings of a perinephric and/or subcapsular hematoma, and *de novo* hematuria (>200 red blood cells per high power field) lasting over 24 hours. Participants needing postbiopsy intervention (*e.g.*, embolization, coiling, explantation, transfusion of packed cells, bladder catheterization or other) were marked having a major bleeding complication. Of the selected patients, 26 were chronically medicated with aspirin—all but one ceased aspirin use 5 days before biopsy. Patients who reported aspirin use had a significantly higher risk of bleeding complications in comparison with those without aspirin exposure (OR=3.19) (see Table 1).

Schwarz *et al.*^20^ performed 1171 protocol biopsies and 499 indication biopsies. The authors aimed to evaluate the safety and adequacy of protocol biopsies after kidney transplantation. Bleeding outcomes included gross hematuria and perirenal hematoma. Patients under sustained low-dose (100 mg) aspirin exposure (n=234) had slightly more often gross hematuria and perirenal hematoma than patients without (gross hematuria, 5.8% versus 3%, *P*=0.046; perirenal hematoma, 3.2% versus 2.2%, not significant). Other aspirin cessation dates were not considered for analysis.

#### Native Kidney Biopsies

The selected studies on native kidney biopsies included a total of 5887 procedures. The association between bleeding events and aspirin use was inconsistent.

Monahan *et al.*^16^ analyzed 2204 native kidney core biopsies. Thirty-one percent of the patients had taken aspirin within 10 days of the biopsy. Only major adverse bleeding events were included in the analysis (Common Terminology Criteria for Adverse Events [CTCAE] grade 3 or higher). Specifically, a grade 3 event includes the need for blood transfusion or radiologic, endoscopic, or elective operative intervention. A grade 4 event has life-threatening consequences and needs urgent intervention. A grade 5 event is death. The authors found no significant association between aspirin exposure and bleeding complications (OR=0.778, *P*-value=0.732 for 325 mg; OR=0.612, *P*-value=0.222 for any dosage).

Lees *et al.*^17^ analyzed 2563 biopsies, including 1499 elective and 1064 emergency procedures. Three hundred twenty-seven patients reported aspirin use (75 mg) and were allowed to continue the drug despite the biopsy. Both minor (patients with hemoglobin drops >2 g/L, visible hematuria, requirement for catheter/irrigation, documented hematoma, additional period of observation or loin pain) and major (patients requiring blood transfusion, surgical or radiological intervention or death) bleeding complications were reported. However, analysis was limited to major bleeding events, and the authors admitted that minor bleeding events may have been underreported. No association was found between aspirin use and major bleeding complications (2.5% versus 1.6%, *P*-value=0.47). In patients taking aspirin, there was no significant increase in the risk of a hemoglobin decrease of >2 g/dl (2.4% versus 2.2%, *P*-value=0.79) or other morbidities (1.5% versus 3.0%, *P*-value=0.15). However, the authors found a significantly increased risk of major bleeding in emergency biopsies compared with elective procedures (3.4% versus 1.1%, *P*-value<0.001).

Mackinnon *et al.*^15^ included two centers with different policies regarding aspirin use in patients undergoing kidney biopsy. One thousand one hundred twenty biopsies were retrospectively analyzed. In one center (n=523), all patients using antiplatelet agents (n=60) were asked to cease their use 5 days before the biopsy. In the other center (n=597), patients using antiplatelet agents (n=75) maintained the medication. Both minor (defined as a fall in hemoglobin of ≥1.0 g/dl, without the need for transfusion or intervention) and major (patients requiring postbiopsy intervention—including transfusions, embolization, or placement of an irrigation catheter) bleeding complications were analyzed. The risk of a hemoglobin drop greater than 1.0 g/dl was significantly higher in the center where patients were kept under antiplatelet agents (31% versus 11.7%; *P*-value=0.008). However, no association was found between concurrent antiplatelet agent use at the time of the biopsy and major complications (1.3% versus 0%; *P*-value=0.56) (see Table 1).

### Native and Graft Kidney Biopsies

The selected studies regarding both native and graft kidney biopsies included 19,421 biopsies.

Atwell *et al.*^14^ retrospectively analyzed 15,181 percutaneous biopsies, including 5832 kidney biopsies—1270 of which were performed under aspirin exposure, defined as the use of aspirin (81 or 325 mg) within 10 days of the biopsy. Only major bleeding events were included in the analysis (CTCAE grade 3 or higher). The authors found no significant difference on the bleeding risk of patients with documented aspirin exposure (1.0% versus 0.6%, *P*-value=0.53) (Table 1).

Potretzke *et al.*^19^ retrospectively analyzed 3245 native kidney and 8105 graft biopsies. Aspirin exposure was categorized according to the dose and cessation date. Last intake dates were divided into the following categories: 8–10 days before the biopsy, 4–7 days before the biopsy, 1–3 days before the biopsy, and the day of the procedure. The authors found that, for any studied dose, usage within 3 days of the biopsy was associated with an increased bleeding risk (OR=3.66, *P*-value <0.001)—this was particularly evident in the group treated with the highest aspirin dosage (325 mg). Only major bleeding events were included in the analysis (CTCAE grade 3 or higher). Aspirin use on the day of the biopsy was shown to have a particular effect on the incidence of postbiopsy bleeding complications (OR=12.4, *P*-value<0.001). Other time intervals for aspirin exposure were not associated with a higher risk of bleeding after the biopsy.

Bonani *et al.*^21^ retrospectively analyzed 733 native kidney and 1506 graft biopsies. The authors aimed to evaluate the safety of kidney biopsies when performed as an outpatient procedure. All bleeding events were reported, but the influence of aspirin use was only evaluated for major bleeding events. Thirty-nine patients were submitted to kidney biopsy under concurrent aspirin use. An undefined number of patients discontinued antiplatelet therapy a week before the biopsy and were considered unexposed to aspirin. Univariate analysis showed an increased risk of major hemorrhage in patients under aspirin (10.3 versus 1.8%; *P*<0.001). Multifactorial logistic regression was performed to identify independent predictors of bleeding. Two models were used: one incorporating only clinical parameters that were available for most patients (model 1) and one incorporating laboratory parameters and blood pressure, which were available for only a subset of patients (model 2). Aspirin use was significantly associated with major bleeding in model 1, but lost significance in model 2.

### Quantitative Synthesis

#### Major Bleeding Events

Owing to significant heterogeneity in methodology, definitions of aspirin exposure, and reported outcome measures, only four^11,13,17,21^ of the ten included studies presented data considered compatible with statistical combination.

**Figure 4:**
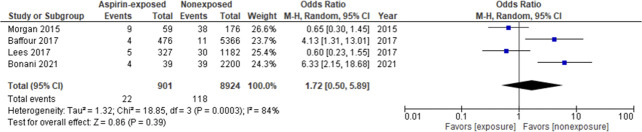
**Forest plot for the major bleeding events between aspirin-exposed and nonexposed groups.** CI, confidence interval.

The primary analysis is described in Figure 4. The aspirin-exposed group included 901 kidney biopsies, and the nonexposed group included 8924 biopsies. The summary OR of major bleeding events was 1.72 (95% CI 0.50 to 5.89, *P*=0.39) with significant heterogeneity among studies (I^2^=84%; *P*=0.0003).

**Figure 5:**
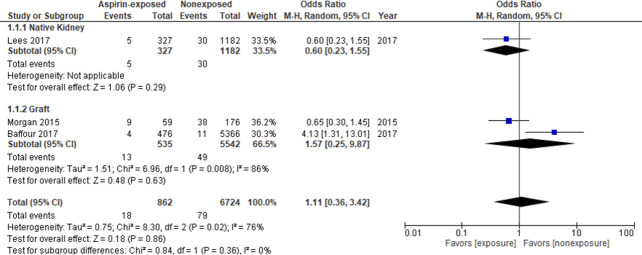
**Subgroup analysis for major bleeding events between aspirin-exposed and nonexposed groups based on kidney type (native or graft).** CI, confidence interval.

Sensitivity analyses were performed to explore potential sources of heterogeneity, including removal of studies with a non–low risk of bias for each domain assessed and the consecutive removal of each individual study (see Supplemental Tables 1 and 2). No significant difference in the overall effect estimates was found. Further subgroup analysis on the basis of the kidney type (native or graft) also showed no significant effect on the pooled estimate and 95% CI (Figure 5). The funnel plot did not show any obvious asymmetry (Supplemental Figure 1).

## Discussion

This study was designed to ascertain the risk of bleeding complications associated with aspirin exposure in patients subjected to PKB. Ten studies fulfilled eligibility criteria, and an aggregate 34,067 kidney biopsies were included. Our meta-analysis showed no significant differences regarding major bleeding events between the aspirin-exposed and nonexposed groups (OR 1.72; 95% CI 0.50 to 5.89, I^2^=84%).

Kumar *et al.*^25^ also reviewed data on the risk of PKB complications under aspirin use. Despite a wider set of inclusion criteria, with guidelines and systematic reviews included in their analysis, the authors lamented the lack of quality evidence and supported the guideline recommending a 7 to 10-day washout period in most elective procedures. However, this review did not include the more recent studies by Baffour *et al.*^13^ and Potretzke *et al.*^19^ with timeframe-dependent subgroup analysis. The inclusion of these two articles is a major strength of our study.

From the selected articles, considering their design and limitations, Baffour *et al.*^13^ and Potretzke *et al.*^19^ were found to present more robust data, particularly because of the inclusion of a timeframe-dependent subgroup analysis. In these studies, aspirin use on the day of the biopsy, particularly in higher dosages, was found to facilitate postbiopsy bleeding complications. However, aspirin cessation 3 days before PKB was found equivalent to more conservative strategies, with no increased risk of bleeding events. Lees *et al.*^17^ and Mackinnon *et al.*^15^ reported no significant association between aspirin use and major bleeding complications in native kidney biopsies, despite an apparent trend in the latter study toward more complications under antiplatelet agent exposure that could arguably become significant with a greater sample size. This trend was also apparent in our meta-analysis, but owing to the high heterogeneity among the enrolled studies and the uncertainty associated with the wider CI, the presented results should be interpreted with great caution.

Evidence supporting deferred aspirin withdrawal is also relevant because of the increased risk of cardiovascular complications found after aspirin interruption,^40–47^ partly explained by rebound phenomena (*e.g.*, increased thromboxane synthesis).^41,48^ This is particularly relevant in patients with established cardiovascular disease where aspirin is used for secondary prevention. The risk of myocardial infarction may increase up to 60% in this population,^49^ and acute coronary syndrome may occur in less than two weeks from the withdrawal of aspirin.^50^ This average delay between aspirin withdrawal and myocardial infarction is consistent with the rebound in platelet activity after aspirin cessation.^33^ Surprisingly, the outcomes of patients who withdrew aspirin are worse than users or previously nonusers, with an increased risk of death.^50^ Shorter aspirin cessation strategies may reduce cardiovascular events because of a more limited aspirin-free window, additionally benefitting from a quicker indication-to-intervention interval, meaning the diagnostic intervention is performed sooner, thus anticipating the introduction of subsequent therapeutic measures. In fact, there is a 30% recovery of platelet function 48 hours after aspirin administration,^31^ which could be enough to ensure a clinically relevant bleeding risk reduction.

The lack of consistent data on the complications of kidney biopsy associated with aspirin leads us to speculate that its periprocedural management should be performed individually, considering clinical and technical aspects. The threshold for aspirin withdrawal is likely to be different in patients who combine good anatomical features and high cardiovascular risk compared with those with an anatomically demanding kidney and low cardiovascular risk. In addition, other aspects, such as the quality of the US image, can also modify the risk of complications and consequently influence the decision to discontinue aspirin. All these individual conditions are difficult to incorporate into guideline recommendations.

Our study presents several limitations. First, most of the included studies had an observational and retrospective design. Second, significant heterogeneity in methodology, participant selection, and reported outcome measures limited the quantitative comparisons. Heterogeneity was particularly evident in the criteria used for aspirin exposure, varying from a confirmed dose the same day as the kidney biopsy^15,17^ to any confirmed intake within 10 days of the intervention,^14,16^ with some authors failing to define relevant aspirin exposure.^11^ Moreover, some studies^11,13,14,16,19^ focused primarily on major adverse bleeding events, meaning minor bleeding complications related to aspirin use may have been underreported.

Additional well-designed prospective controlled trials are needed for a better comprehensive understanding of this association. For future research, we suggest authors should focus not only on the bleeding complications related to aspirin exposure but also on the clinical outcomes associated with aspirin discontinuation, namely thrombotic complications and cardiovascular events. This would allow clinicians to make better informed decisions with a more substantiated understanding of the risks and benefits of aspirin cessation.

In patients awaiting PKB, the best strategy regarding aspirin cessation is still undetermined because of lack of high-quality evidence. Our meta-analysis did not show a significantly increased risk of major bleeding complications in aspirin-exposed patients.

## Supplementary Material

SUPPLEMENTARY MATERIAL

## Supplemental Material

Supplemental Table 1. Sensitivity analyses after the removal of studies with a non–low risk of bias for each domain.

Supplemental Table 2. Sensitivity analyses after the consecutive removal of individual trials.

Supplemental Figure 1. Funnel plot for the assessment of publication bias.

## References

[1] Hogan JJ Mocanu M Berns JS. The native kidney biopsy: update and evidence for best practice. Clin J Am Soc Nephrol. 2016;11(2):354-362. doi:10.2215/CJN.05750515 26339068 PMC4741037

[2] Stratta P Canavese C Marengo M Mesiano P Besso L Quaglia M. Risk management of renal biopsy: 1387 cases over 30 years in a single centre. Eur J Clin Invest. 2007;37(12):954-963. doi:10.1111/j.1365-2362.2007.01885.x 18036029

[3] Nayak-Rao S. Percutaneous native kidney biopsy in patients receiving antiplatelet agents—is it necessary to stop them routinely? Indian J Nephrol. 2015;25(3):129-132. doi:10.4103/0971-4065.147374 26060359 PMC4446914

[4] Hergesell O Felten H Andrassy K Kühn K Ritz E. Safety of ultrasound-guided percutaneous renal biopsy-retrospective analysis of 1090 consecutive cases. Nephrol Dial Transplant. 1998;13(4):975-977. doi:10.1093/ndt/13.4.975 9568860

[5] Eiro M Katoh T Watanabe T. Risk factors for bleeding complications in percutaneous renal biopsy. Clin Exp Nephrol. 2005;9(1):40-45. doi:10.1007/s10157-004-0326-7 15830272

[6] Manno C Strippoli GF Arnesano L Bonifati C Campobasso N Gesualdo L. Predictors of bleeding complications in percutaneous ultrasound-guided renal biopsy. Kidney Int. 2004;66(4):1570-1577. doi:10.1111/j.1523-1755.2004.00922.x 15458453

[7] Shidham GB Siddiqi N Beres JA Logan B Nagaraja HN Shidham SG. Clinical risk factors associated with bleeding after native kidney biopsy. Nephrology (Carlton). 2005;10(3):305-310. doi:10.1111/j.1440-1797.2005.00394.x 15958047

[8] Poggio ED McClelland RL Blank KN Hansen S Bansal S Bomback AS. Systematic review and meta-analysis of native kidney biopsy complications. Clin J Am Soc Nephrol. 2020;15(11):1595-1602. doi:10.2215/CJN.04710420 33060160 PMC7646247

[9] Furness PN Philpott CM Chorbadjian MT Nicholson ML Bosmans JL Corthouts BL. Protocol biopsy of the stable renal transplant: a multicenter study of methods and complication rates. Transplantation. 2003;76(6):969-973. doi:10.1097/01.tp.0000082542.99416.11 14508363

[10] Korbet SM Volpini KC Whittier WL. Percutaneous renal biopsy of native kidneys: a single-center experience of 1,055 biopsies. Am J Nephrol. 2014;39(2):153-162. doi:10.1159/000358334 24526094

[11] Morgan TA Chandran S Burger IM Zhang CA Goldstein RB. Complications of ultrasound-guided renal transplant biopsies. Am J Transplant. 2016;16(4):1298-1305. doi:10.1111/ajt.13622 26601796

[12] Trajceska L Severova-Andreevska G Dzekova-Vidimliski P Nikolov I Selim G Spasovski G. Complications and risks of percutaneous renal biopsy. Open Access Maced J Med Sci. 2019;7(6):992-995. doi:10.3889/oamjms.2019.226 30976347 PMC6454172

[13] Baffour FI Hickson LJ Stegall MD Dean PG Gunderson TM Atwell TD. Effects of aspirin therapy on ultrasound-guided renal allograft biopsy bleeding complications. J Vasc Interv Radiol. 2017;28(2):188-194. doi:10.1016/j.jvir.2016.10.021 27993506 PMC5258683

[14] Atwell TD Smith RL Hesley GK Callstrom MR Schleck CD Harmsen WS. Incidence of bleeding after 15,181 percutaneous biopsies and the role of aspirin. AJR Am J Roentgenol. 2010;194(3):784-789. doi:10.2214/ajr.08.2122 20173160

[15] Mackinnon B Fraser E Simpson K Fox JG Geddes C. Is it necessary to stop antiplatelet agents before a native renal biopsy? Nephrol Dial Transplant. 2008;23(11):3566-3570. doi:10.1093/ndt/gfn282 18503099

[16] Monahan H Gunderson T Greene E Schmit G Atwell T Schmitz J. Risk factors associated with significant bleeding events after ultrasound-guided percutaneous native renal biopsies: a review of 2204 cases. Abdom Radiol (NY). 2019;44(6):2316-2322. doi:10.1007/s00261-019-01962-z 30830293

[17] Lees JS McQuarrie EP Mordi N Geddes CC Fox JG Mackinnon B. Risk factors for bleeding complications after nephrologist-performed native renal biopsy. Clin Kidney J. 2017;10(4):573-577. doi:10.1093/ckj/sfx012 28852497 PMC5570080

[18] Kuiper GJAJ Christiaans MHL Mullens MHJM Ten Cate H Hamulýak K Henskens YMC. Routine haemostasis testing before transplanted kidney biopsy: a cohort study. Transplant Int. 2018;31(3):302-312. doi:10.1111/tri.13090 29108097

[19] Potretzke TA Harvey JA Gunderson TM Jensen NM Schmit GD McBane RD. Frequency of bleeding complications after percutaneous core needle biopsy and the association with aspirin usage and length of aspirin discontinuation. AJR Am J Roentgenol. 2019;213(1):211-215. doi:10.2214/AJR.18.20366 30995091

[20] Schwarz A Gwinner W Hiss M Radermacher J Mengel M Haller H. Safety and adequacy of renal transplant protocol biopsies. Am J Transplant. 2005;5(8):1992-1996. doi:10.1111/j.1600-6143.2005.00988.x 15996250

[21] Bonani M Seeger H Weber N Lorenzen JM Wüthrich RP Kistler AD. Safety of kidney biopsy when performed as an outpatient procedure. Kidney Blood Press Res. 2021;46(3):310-322. doi:10.1159/000515439 34077930

[22] Wilczek HE. Percutaneous needle biopsy of the renal allograft. A clinical safety evaluation of 1129 biopsies. Transplantation. 1990;50(5):790-797. doi:10.1097/00007890-199011000-00010 2238054

[23] Halimi JM Gatault P Longuet H Barbet C Bisson A Sautenet B. Major bleeding and risk of death after percutaneous native kidney biopsies: a French Nationwide cohort study. Clin J Am Soc Nephrol. 2020;15(11):1587-1594. doi:10.2215/CJN.14721219 33060158 PMC7646233

[24] Brachemi S Bollée G. Renal biopsy practice: what is the gold standard? World J Nephrol. 2014;3(4):287-294. doi:10.5527/wjn.v3.i4.287 25374824 PMC4220363

[25] Kumar V Mitchell MD Umscheid CA Berns JS Hogan JJ. Risk of complications with use of aspirin during renal biopsy: a systematic review. Clin Nephrol. 2018;89(2):67-76. doi:10.5414/cn109274 29319492

[26] Lees JS McQuarrie EP Mackinnon B. Renal biopsy: it is time for pragmatism and consensus. Clin Kidney J. 2018;11(5):605-609. doi:10.1093/ckj/sfy075 30289128 PMC6165764

[27] Patel IJ Davidson JC Nikolic B Salazar GM Schwartzberg MS Walker TG. Consensus guidelines for periprocedural management of coagulation status and hemostasis risk in percutaneous image-guided interventions. J Vasc Interv Radiol. 2012;23(6):727-736. doi:10.1016/j.jvir.2012.02.012 22513394

[28] Morais J Aspirina MSP. 120 anos de história: para além da prevenção cardiovascular. 1^a^ ed. Heartbrain; 2018.

[29] O'Brien CW Juraschek SP Wee CC. Prevalence of aspirin use for primary prevention of cardiovascular disease in the United States: results from the 2017 National Health Interview Survey. Ann Intern Med. 2019;171(8):596-598. doi:10.7326/m19-0953 31330542 PMC7251544

[30] Fang J George MG Hong Y Loustalot F. Use of aspirin for prevention of recurrent atherosclerotic cardiovascular disease among adults—20 States and the District of Columbia, 2013. MMWR Morb Mortal Wkly Rep. 2015;64(27):733-737. PMID:26182190 26182190 PMC4584583

[31] Hall R Mazer CD. Antiplatelet drugs: a review of their pharmacology and management in the perioperative period. Anesth Analg. 2011;112(2):292-318. doi:10.1213/ane.0b013e318203f38d 21212258

[32] Patrignani P Filabozzi P Patrono C. Selective cumulative inhibition of platelet thromboxane production by low-dose aspirin in healthy subjects. J Clin Invest. 1982;69(6):1366-1372. doi:10.1172/jci110576 7045161 PMC370209

[33] FitzGerald GA Oates JA Hawiger J Maas RL Roberts LJ Lawson JA. Endogenous biosynthesis of prostacyclin and thromboxane and platelet function during chronic administration of aspirin in man. J Clin Invest. 1983;71(3):676-688. doi:10.1172/jci110814 6338043 PMC436917

[34] Bell AD Roussin A Cartier R Chan WS Douketis JD Gupta A. The use of antiplatelet therapy in the outpatient setting: Canadian Cardiovascular Society Guidelines. Can J Cardiol. 2011;27(3):S1-S59. doi:10.1016/j.cjca.2010.12.015 21640290

[35] Douketis JD Spyropoulos AC Spencer FA Mayr M Jaffer AK Eckman MH. Perioperative management of antithrombotic therapy: antithrombotic therapy and prevention of thrombosis, 9th ed: American College of Chest Physicians evidence-based clinical practice guidelines. Chest. 2012;141(2):e326S-e350S. doi:10.1378/chest.11-2298 22315266 PMC3278059

[36] MacGinley R Champion De Crespigny PJ Gutman T Lopez-Vargas P Manera K Menahem S. KHA-CARI Guideline recommendations for renal biopsy. Nephrology (Carlton). 2019;24(12):1205-1213. doi:10.1111/nep.13662 31490584

[37] Higgins JP Thompson SG. Quantifying heterogeneity in a meta-analysis. Stat Med. 2002;21(11):1539-1558. doi:10.1002/sim.1186 12111919

[38] Reschen ME Mazzella A Sharples E. A retrospective analysis of the utility and safety of kidney transplant biopsies by nephrology trainees and consultants. Ann Med Surg (Lond). 2018;28:6-10. doi:10.1016/j.amsu.2018.02.001 29552340 PMC5852268

[39] Ori Y Neuman H Chagnac A Siegal A Tobar A Itkin M. Using the automated biopsy gun with real-time ultrasound for native renal biopsy. Isr Med Assoc J. 2002;4(9):698-701. PMID:12440234 12440234

[40] Andrulli S Rossini M Gigliotti G La Manna G Feriozzi S Aucella F. The risks associated with percutaneous native kidney biopsies: a prospective study. Nephrol Dial Transplant. 2022;38(3):655-663. doi:10.1093/ndt/gfac177 PMC9976765 35587882

[41] Burger W Chemnitius JM Kneissl GD Rücker G. Low-dose aspirin for secondary cardiovascular prevention—cardiovascular risks after its perioperative withdrawal versus bleeding risks with its continuation—review and meta-analysis. J Intern Med. 2005;257(5):399-414. doi:10.1111/j.1365-2796.2005.01477.x 15836656

[42] Murphy GJ Taha R Windmill DC Metcalfe M Nicholson ML. Influence of aspirin on early allograft thrombosis and chronic allograft nephropathy following renal transplantation. Br J Surg. 2002;88(2):261-266. doi:10.1046/j.1365-2168.2001.01671.x 11167878

[43] Robertson AJ Nargund V Gray DW Morris PJ. Low dose aspirin as prophylaxis against renal-vein thrombosis in renal-transplant recipients. Nephrol Dial Transplant. 2000;15(11):1865-1868. doi:10.1093/ndt/15.11.1865 11071979

[44] Samama CM Bastien O Forestier F Denninger MH Isetta C Juliard JM. Antiplatelet agents in the perioperative period: expert recommendations of the French Society of Anesthesiology and Intensive Care (SFAR) 2001—summary statement. Can J Anaesth. 2002;49(6):S26-S35. 12557412

[45] Biondi-Zoccai GG Lotrionte M Agostoni P Abbate A Fusaro M Burzotta F. A systematic review and meta-analysis on the hazards of discontinuing or not adhering to aspirin among 50,279 patients at risk for coronary artery disease. Eur Heart J. 2006;27(22):2667-2674. doi:10.1093/eurheartj/ehl334 17053008

[46] Maulaz AB Bezerra DC Michel P Bogousslavsky J. Effect of discontinuing aspirin therapy on the risk of brain ischemic stroke. Arch Neurol. 2005;62(8):1217-1220. doi:10.1001/archneur.62.8.1217 16087761

[47] Ferrari E Benhamou M Cerboni P Marcel B. Coronary syndromes following aspirin withdrawal: a special risk for late stent thrombosis. J Am Coll Cardiol. 2005;45(3):456-459. doi:10.1016/j.jacc.2004.11.041 15680728

[48] Vial JH McLeod LJ Roberts MS. Rebound elevation in urinary thromboxane B2 and 6-keto-PGF1 alpha excretion after aspirin withdrawal. Adv Prostaglandin Thromboxane Leukot Res. 1991;21A:157-160. PMID:1825534 1825534

[49] Rodríguez LA Cea-Soriano L Martín-Merino E Johansson S. Discontinuation of low dose aspirin and risk of myocardial infarction: case-control study in UK primary care. BMJ. 2011;343:d4094. doi:10.1136/bmj.d4094 21771831 PMC3139911

[50] Collet JP Montalescot G Blanchet B Tanguy ML Golmard JL Choussat R. Impact of prior use or recent withdrawal of oral antiplatelet agents on acute coronary syndromes. Circulation. 2004;110(16):2361-2367. doi:10.1161/01.cir.0000145171.89690.b4 15477397

