# Supplementary material for: Effects of Aspirin on Kidney Biopsy Bleeding Complications: A Systematic Review and Meta-Analysis (PROSPERO 2021 CRD42021261005)
Source: Kidney360. 2023 Mar 23;4(5):700–10. doi: 10.34067/KID.0000000000000091 (PMC10278841; doi:10.34067/KID.0000000000000091)

Supplemental Material

Table of Contents

Table S1 – Sensitivity analyses after the removal of studies with a non-low risk of bias for each domain.

Table S2 – Sensitivity analyses after the consecutive removal of individual trials.

Figure S1 – Funnel plot for assessment of publication bias.

Table S1 - Sensitivity analyses after the removal of studies with a non-low risk of bias for each domain.

Excluding studies with non-low risk of bias of each domain	Odds Ratio, 95% confidence interval	I^2
Confounding bias	Not estimable	Not estimable
Selection bias	1.72 [0.50, 5.89]	84%
Classification bias	1.72 [0.50, 5.89]	84%
Performance bias	2.48 [0.52, 11.92]	86%
Missing data	Not estimable	Not estimable
Measurement bias	Not estimable	Not estimable
Reporting bias	1.53 [0.22, 10.83]	85%

Table S2- Sensitivity analyses after the consecutive removal of individual trials.

Study removed	Odds Ratio 95% confidence interval	I^2	Test for overall Effect (p-value)
Baffour 2017	1.31 [0.31, 5.52]	86%	P=0.71
Bonani 2021	1.11 [0.36, 3.42]	76%	P=0.86
Lees 2017	2.48 [0.52, 11.92]	86%	P=0.26
Morgan 2015	2.45 [0.49, 12.16]	85%	P=0.27

Figure S1- Funnel plots for assessment of publication bias.

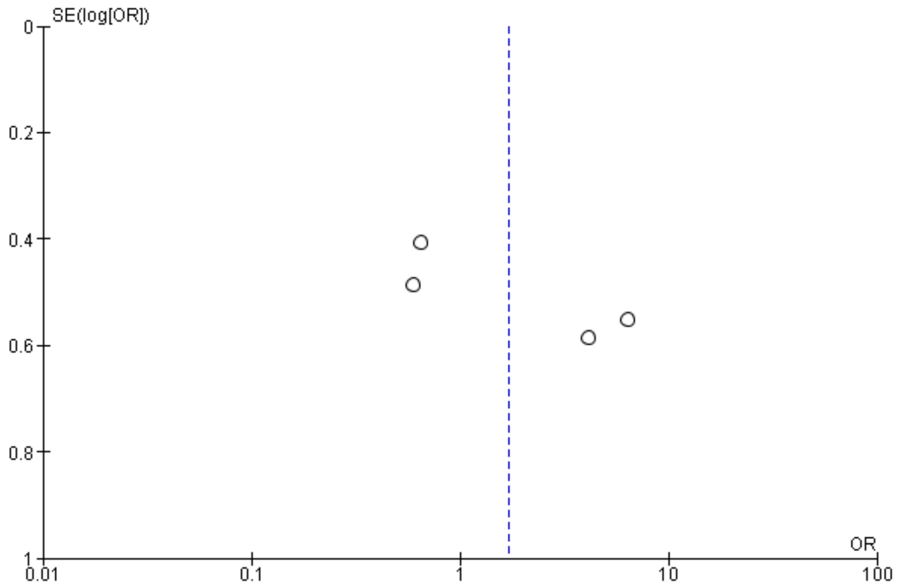

Supplement: SUPPLEMENTARY MATERIAL [file kidney360-4-700-s001.pdf]
